# Crocin ameliorates neuroinflammation and cognitive impairment in mice with Alzheimer's disease by activating PI3K/AKT pathway

**DOI:** 10.1002/brb3.3503

**Published:** 2024-05-22

**Authors:** Wenwen Su, Yanbo Wang, Sen Shao, Xiaojun Ye

**Affiliations:** ^1^ Department of Internal Medicine CiXi Seventh People's Hospital Ningbo Zhejiang China; ^2^ Department of Neurology The Third Affiliated Hospital of Zhejiang Chinese Medicine University Hangzhou Zhejiang China; ^3^ Department of Neurology The Xixi Hospital of Hangzhou Affiliated to Zhejiang University School of Medicine Hangzhou Zhejiang China; ^4^ Department of Neurology The Affiliated Hospital of Hangzhou Normal University Hangzhou Zhejiang China

**Keywords:** Alzheimer's disease, Crocin, inflammation, pi3k/akt signaling

## Abstract

**Background:**

Crocin has a good prospect in the treatment of Alzheimer's disease (AD), but the mechanisms underlying its neuroprotective effects remain elusive. This study aimed to investigate the neuroprotective effects of Crocin and its underlying mechanisms in AD.

**Methods:**

AD mice were set up by injecting Aβ_25‐35_ solution into the hippocampus. Then, the AD mice were injected intraperitoneally with 40 mg/kg/day of Crocin for 14 days. Following the completion of Crocin treatment, an open‐field test, Y‐maze test and Morris water maze test were conducted to evaluate the impact of Crocin on spatial learning and memory deficiency in mice. The effects of Crocin on hippocampal neuron injury, proinflammatory cytokine expressions (IL‐1β, IL‐6, and TNF‐α), and PI3K/AKT signaling‐related protein expressions were measured using hematoxylin and eosin staining, Western blot, and quantitative real‐time polymerase chain reaction (qRT‐PCR) experiments, respectively.

**Results:**

Crocin attenuated Aβ_25‐35_‐induced spatial learning and memory deficiency and hippocampal neuron injury. Furthermore, the Western blot and qRT‐PCR results showed that Crocin effectively suppressed inflammation and activated the PI3K/AKT pathway in Aβ_25‐35_‐induced mice.

**Conclusion:**

Crocin restrained neuroinflammation via the activation of the PI3K/AKT pathway, thereby ameliorating the cognitive dysfunction of AD mice.

## Graphical abstract

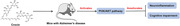

## 1 INTRODUCTION

Alzheimer's disease (AD) is characterized by degeneration of the central nervous system, and its onset process is insidious (Lane et al., 2018). Early detection of AD can be challenging, and in the later stages, patients become agitated and aggressive, accompanied by sleep disorders and loss of reasoning ability (Robakis, 2010). AD is the most prevalent form of dementia, accounting for 60%−70% of the total dementia cases (2021 Alzheimer's disease facts and figures). The incidence of AD in China gradually increases with age, and the explicit and implicit medical burdens on patients and their families are also becoming heavier (Lei et al., 2021). Abnormal aggregation of Aβ is crucial in the pathogenesis of AD, Aβ can lead to the phosphorylation of Tau neuronal protein, induce synaptic loss and apoptosis of neurons, and ultimately affect cognitive function (Sardar Sinha et al., 2018). Inflammation also plays a fundamental role in AD. Studies have shown that patients with AD have elevated levels of inflammatory markers (Gagliardi et al., 2022). Additionally, inhibiting inflammation in AD has been found to slow cognitive decline (Nillert et al., 2022). Current therapies for AD mainly focus on psychological intervention, environmental behavior improvement, and drug treatment for AD, but their effectiveness is limited (Soria Lopez et al., 2019). Therefore, while continuing to actively develop drugs targeting Aβ, it has become a key strategy for AD treatment to further explore the mechanisms of Aβ action.

Traditional Chinese medicine is often used in the treatment of AD due to its advantages of multitarget, multisystem, and multichannel, which align with the characteristics of the complex pathological mechanisms of AD (Chen et al., 2020). Saffron, a traditional medicinal material in China, contains pharmacological components that have neuroprotective, antidepressant, cardioprotective, and memory‐enhancing effects (Hosseini et al., 2018). Crocin is the most important pharmacological active component of saffron, which has neuroprotective effects in Parkinson's Drosophila model, as well as in neurobehavioral and neurochemical sequelae caused by methylphenidate, and AD (Ebrahimzadeh et al., 2019; Soeda et al., 2016). Farokhnia et al. (2014) have randomized 68 patients with moderate to severe AD to receive either memantine (20 mg/day) or saffron capsules (30 mg/day); after 12 months of treatment, saffron capsules are found to have the same effect as memantine in delaying the cognitive decline of moderate to severe AD patients, demonstrating a certain therapeutic effect of saffron on AD. In addition, scholars have reported that Crocin can prevent cognitive decline in rats exposed to acute high‐altitude hypoxia by promoting mitochondrial biosynthesis, ameliorating oxidative stress injury, and decreasing neuronal apoptosis (Zhang et al., 2020). However, further research is necessary to fully understand the roles and mechanisms of Crocin in AD.

The PI3K/AKT pathway has a significant impact on the central nervous system affecting the proliferation, differentiation, and death of neurons (Long et al., 2021). In addition, Aβ exposure can directly interrupt PI3K/AKT signaling in the brains of AD patients (Fu et al., 2016). It has been reported that Crocin can inhibit the progression of neurological diseases (such as AD, retinal ischemia/reperfusion (IR) injury, and cerebral infarction) by regulating the PI3K/AKT pathway (Qi et al., 2013; Taheri et al., 2021; Zhao et al., 2022). Nevertheless, the exact effects of Crocin on the PI3K/AKT pathway in AD remain unclear.

Therefore, in this research, we performed in vivo experiments to investigate whether Crocin can ameliorate the cognitive behavior of AD mice by regulating the PI3K/AKT pathway, thus providing a novel approach to treating AD.

## 2 MATERIALS AND METHODS

### 2.1 Animals

Thirty male ICR mice weighing between 23 and 25 g were obtained from Shanghai SLAC Laboratory Animal Co., Ltd. (China). The mice were adaptively fed for 7 days at an ambient temperature of 25 ± 2°C and a humidity of 55 ± 5%. All mice were placed under artificial 12 h/12 h circadian light. All procedures involving animal manipulations were reviewed and ratified by the Institutional Animal Care and Use Committee at the Animal Experimentation Ethics Committee of Zhejiang Eyong Pharmaceutical Research and Development Center.

### 2.2 Establishment of the AD model

Thirty mice were randomized into the sham group, Aβ_25‐35_ group, and Aβ_25‐35_+Crocin group (*n* = 10). Aβ_25‐35_ (A4559, Sigma‐Aldrich, USA) of 1 mL was dissolved in sterile saline (the final concentration of Aβ_25‐35_ solution was 5 µg/µL). The prepared Aβ_25‐35_ solution was incubated at 37°C for 7 days to form aggregates. An AD mouse model was established as previously described (Zhang et al., 2021). All mice were anesthetized with 3% isoflurane (792632, Sigma‐Aldrich, USA), and the brain was fixed using the brain stereotactic apparatus (7100‐R, RWD, China). Aβ_25‐35_ (2 µL) was injected into the hippocampus of mice, specifically, the anteroposterior (AP) was 2.0 mm, the mediolateral (ML) was 2.0 mm, and the dorsoventral (DV) was 1.7 mm. The mice in the sham group were injected with an equivalent volume of sterile saline in the same manner. After 24 h, the mice in the Aβ_25‐35_+Crocin group were injected intraperitoneally with 40 mg/kg of Crocin (purity ≥ 98%, 42553‐65‐1, Shanghai YuanYe Biotechnology Co., Ltd, dissolved in normal saline) daily for 14 consecutive days (Salem et al., 2022; Wang et al., 2024). The other two groups of mice received the same amount of normal saline.

### 2.3 Open‐field test (OFT)

OFT was employed to assess the locomotor activity and anxiety‐related behaviors of the mice after 14 days of treatment (Lu et al., 2019). The OFT device was a square case with a bottom area of 50 × 50 cm. The square case was bright and had a central area of 25 × 25 cm and a peripheral zone. The mice were placed in a fixed central position, and 5‐min mouse movement trajectories and time spent in the central area (latency) were recorded and then analyzed by ANY‐maze^TM^ (Stoelting, USA). After each trial, the chamber was cleaned with 70% ethanol and dried to remove any odor cues.

### 2.4 Y‐maze test

Y‐maze apparatus was selected to measure spontaneous spatial working memory (Pi et al., 2021). The apparatus consisted of three identical arms (40 × 4.5 × 12 cm). There was a food supply at the end of one arm. The mice were placed in one arm and allowed to search for food at the end of one of the other two arms. Search time (latency) was recorded by the camera and analyzed using ANY‐maze^TM^ software (Stoeling Co., USA).

### 2.5 Morris water maze (MWM) test

MWM experiment was applied to test the cognitive ability of mice (Vorhees & Williams, 2006). The water maze was mainly composed of a drum (150 cm in diameter and 62.5 cm in height) and an automatic video analysis system. At the same time, a movable platform with a diameter of 10 cm and a height of 20 cm was placed in the water maze as a platform for the mice. The water temperature was maintained at 22°C−23°C and the depth was 30 cm. The training phase of the positioning navigation experiment was conducted three times per day for five consecutive days, with each training lasting 120 s. During training, the mice were placed into the pool from three water entry points facing the pool wall. This study recorded the latency, expressed in seconds, as the time it took for the mice to find the platform and stand on it after entering the water. Subsequently, on day 6, the space exploration experiment was performed in which the platform was removed from the drum and the mice were placed into the water from the same entry point in the third quadrant. The swimming trajectory and the number of crossing the original platform quadrant within 60 s were recorded.

### 2.6 Hematoxylin and eosin (H&E) staining

H&E staining kits (G1003, Servicebio, China) were used to observe the morphology of hippocampal pyramidal neurons in the study. After completion of the behavioral tests, all animals were euthanized by inhalation of an overdose of CO_2_. The hippocampal tissues were immediately removed and fixed in 4% paraformaldehyde (P804536, Macklin, China). After dehydration, the tissues were embedded in paraffin and 10 µm thick sections were acquired by a pathological slicer (RM2016, Leica, China). Hereafter, the sections were dewaxed and hydrated before being stained with hematoxylin for 5 min. For differentiation of the sections, 1% hydrochloric acid was utilized. After counterstaining with 1% eosin solution, the sections were treated with increasing concentrations of ethanol and subjected to xylene permeabilization for 5 min. Finally, the sections were sealed with neutral balsam (WG10004160, Servicebio, China) and observed under an optical microscope (ECLIPSE 80i, Nikon, Japan).

### 2.7 Quantitative real‐time polymerase chain reaction (QRT‐PCR)

Total RNA from the hippocampal tissues was isolated for cDNA synthesis by applying the RNA extraction kits (LS1040, Promega, USA). Afterward, cDNA synthesis was done with reverse transcription kits (CW2569, CWBIO, China). The reaction system was performed based on the protocol of SYBR Premix Ex TaqII (RR820A, Takara, Japan). A total of 20 µL reactions were conducted via a QuantStudio 5 qPCR machine (Applied Biosystems, USA). β‐actin was used as the normalizer. The 2^−ΔΔCt^ formula was employed for calculating the expressions of genes. The primer sequences used in the study are shown in Table 1.

**TABLE 1:** Primer sequences of the genes.

Gene	Forward primer	Reverse primer
Mouse TNF‐α	CAGGCGGTGCCTATGTCTC	CGATCACCCCGAAGTTCAGTAG
Mouse IL‐1β	GAAATGCCACCTTTTGACAGTG	TGGATGCTCTCATCAGGACAG
Mouse IL‐6	CTGCAAGAGACTTCCATCCAG	AGTGGTATAGACAGGTCTGTTGG
Mouseβ‐actin	CAGCCTTCCTTCTTGGGTAT	GCTCAGTAACAGTCCGCCTA

### 2.8 Western blot

The hippocampal tissues were homogenated using RIPA Buffer (P0013D, Beyotime, China). After centrifugation, the supernatants were collected and the protein concentrations were detected with the BCA kits (pc0020, Solarbio, China). With these steps carried out, the protein was electrophoresed and loaded onto PVDF membranes (10600023, GE Healthcare Life, USA). After sealing, the blocked membranes were reacted with primary antibodies (4°C, all night). Then, the membranes were intervened with an anti‐rabbit secondary antibody (31466, Invitrogen, USA) for another 1 h. Finally, the membranes were visualized with ECL reagents (SQ202, Epizyme, China) and analyzed with ImageJ. The primary antibodies used were phospho‐PI3K p85 alpha (Tyr607, 1:1000, AF3241), PI3K p85 alpha (1:1000, AF6241), phospho‐AKT2 (Ser474, 1:2000, AF3264), AKT2 (1:2000, AF6264), IL‐1β (1:1000, AF5103), IL‐6 (1:1000, DF6087), TNF‐α (1:1000, AF7014), and β‐actin (1:10000, AF7018) were acquired from Affinity (USA).

### 2.9 Statistics

All experiments were conducted in triplicate, and the data of the study were presented as mean ± standard deviation. Statistical analyses and figures were generated by SPSS software (19.0, IBM, USA). One‐way ANOVA with the Tukey's test was exploited to compare the differences of multiple groups. Kruskal–Wallis *H* test was applied for heterogeneity of variance. A *p *< .05 was considered a statistically significant difference.

## 3 RESULTS

### 3.1 Crocin mitigated Aβ25‐35‐mediated hippocampal neuronal injury

**FIGURE 1:**
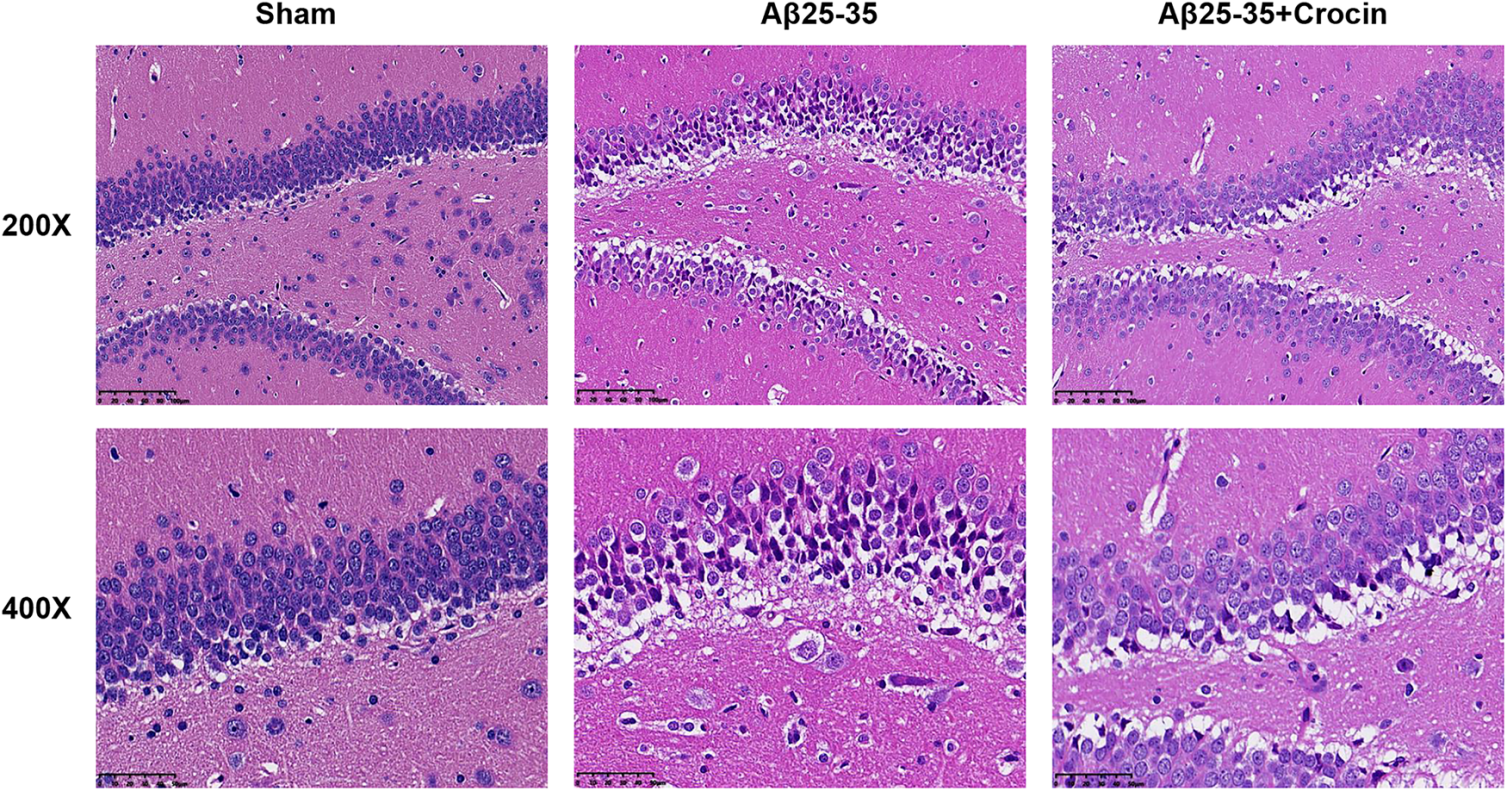
Crocin mitigated Aβ_25‐35_‐mediated hippocampal neuronal injury in Alzheimer's disease mice. The effects of Crocin on hippocampal neuron injury in Aβ_25‐35_‐mediated mice were monitored by hematoxylin and eosin staining (magnification ×200, 400).

The H&E staining assay showed that the pyramidal neurons in the hippocampus of the Aβ_25‐35_ group were arranged sparsely with an incomplete structure. However, in comparison to the Aβ_25‐35_ group, the hippocampal pyramidal neurons of the mice in the Aβ_25‐35_+Crocin group were arranged more closely and had a more intact structure (Figure 1).

### 3.2 Crocin attenuated Aβ25‐35‐induced spatial learning and memory deficiency

**FIGURE 2:**
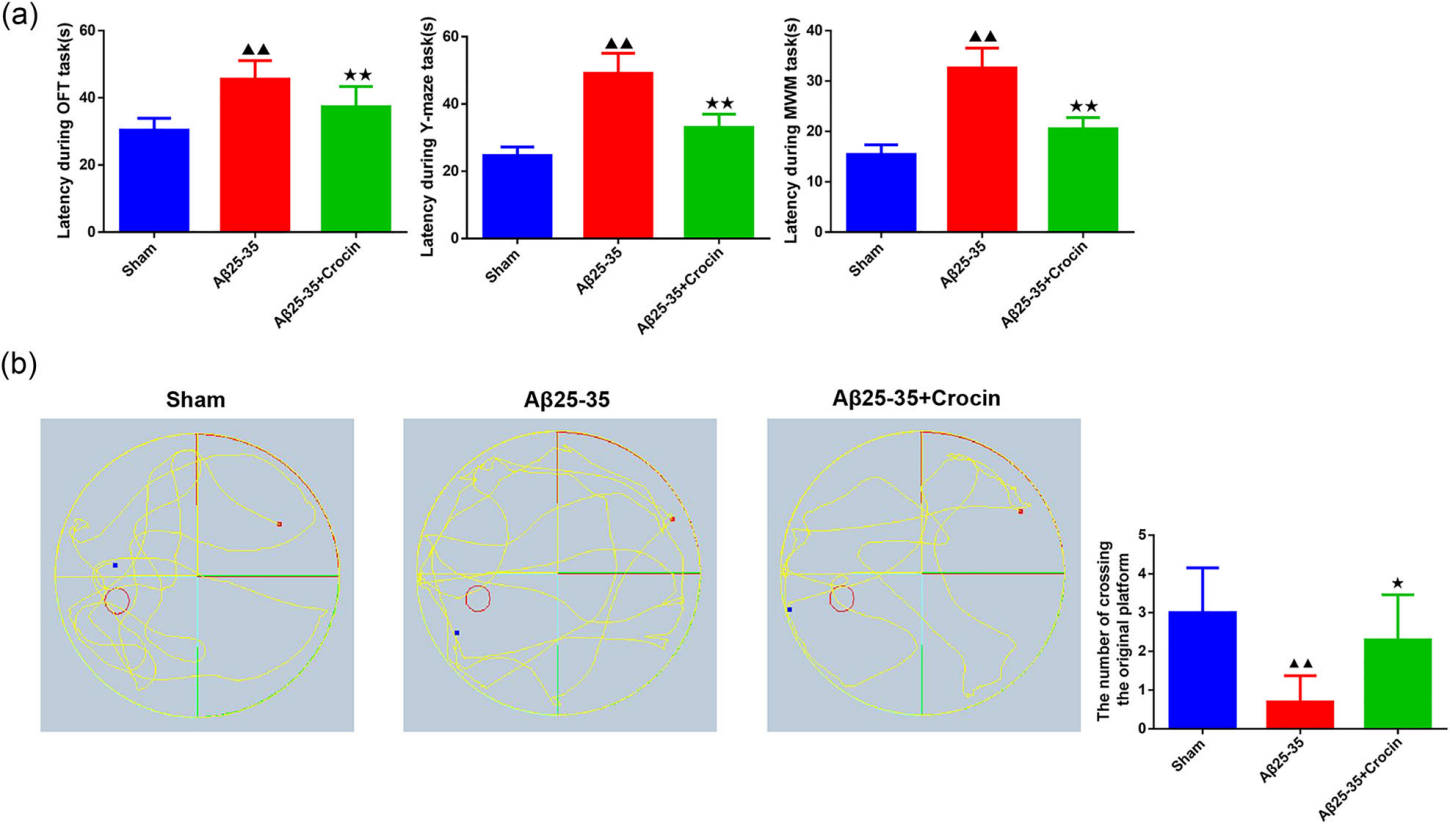
Crocin attenuated Aβ_25‐35_‐induced spatial learning and memory deficiency. **(a)** The effects of Crocin on spatial learning and memory deficiency in Aβ_25‐35_‐mediated mice were examined using the open‐field test (OFT), Y‐maze, and Morris water maze (MWM) tests (*n* = 10). **(b)** Representative swimming trajectory and the number of crossing the original platform in different groups. The red dot indicated the entry point, the blue dot indicated the position of the mice at the end of the experiment, and the red circle was the position of the original platform. ^▲▲^ *p *< .01 vs. sham group; ^★★^ *p *< .01 vs. Aβ_25‐35_ group.

The results of the OFT and Y‐maze tests revealed that Aβ_25‐35_ induction significantly increased the latency during the OFT/Y‐maze task, whereas Crocin intervention significantly reduced the time spent in the center during the OFT and the time spent searching for food in the Y‐maze task for AD mice (Figure 2a, *p *< .01). Meanwhile, there was an increase in latency during the MWM task in the Aβ_25‐35_ group compared to the sham group (Figure 2a, *p *< .01). However, Crocin treatment significantly offset the latency during the MWM task modulated by Aβ_25‐35_ (Figure 2a, *p *< .01). The results of the space exploration experiment in the MWM task showed that relative to the sham group, mice in the Aβ_25‐35_ group significantly decreased the number of crossing the original platform. Nevertheless, Crocin treatment significantly reversed this situation (Figure 2b, *p *< .01).

### 3.3 Crocin suppressed inflammation and activated PI3K/AKT signaling in hippocampal tissues of AD mice

**FIGURE 3:**
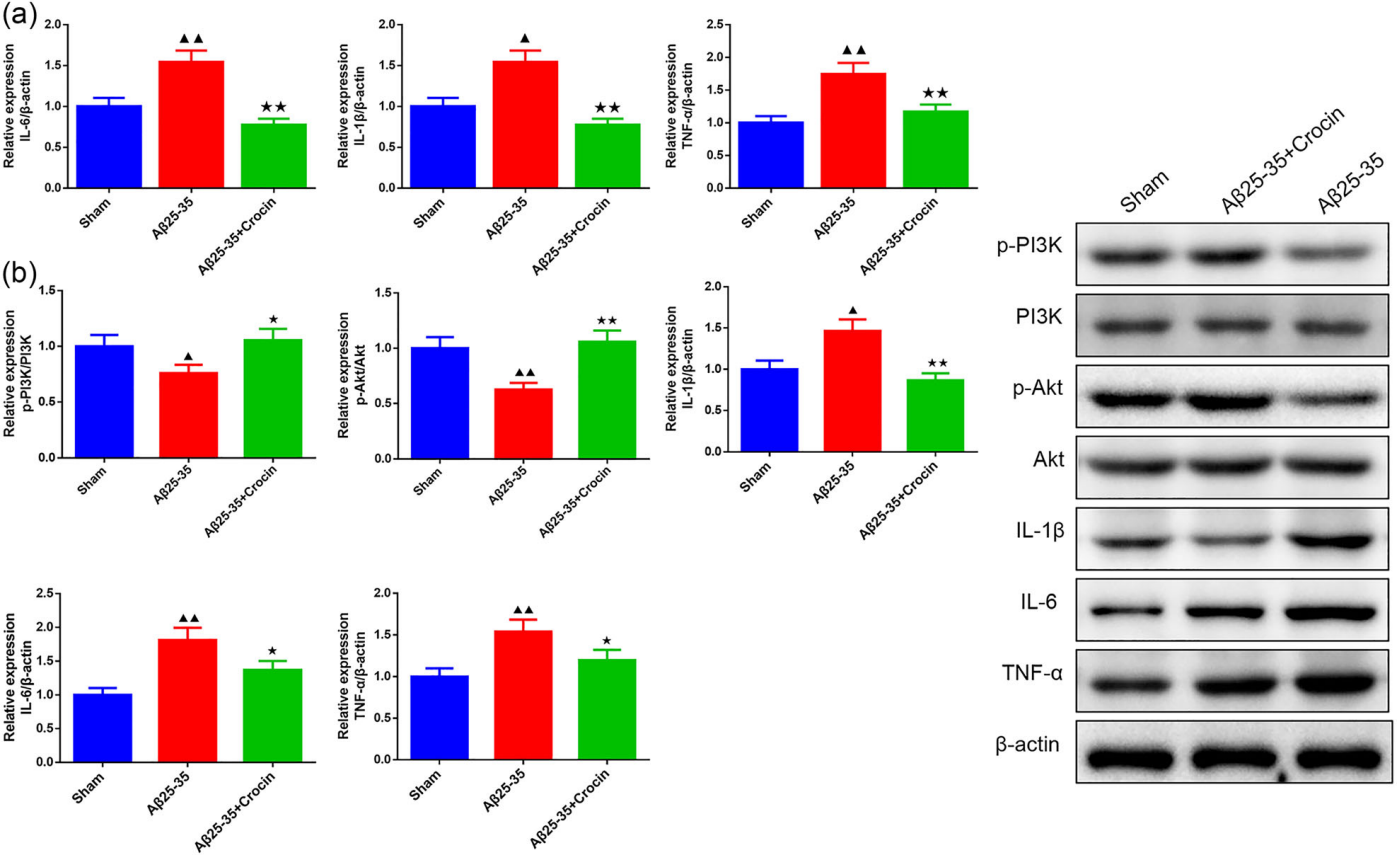
Crocin suppressed inflammation and activated PI3K/AKT signaling of the hippocampal tissues in AD mice. **(a)** The inflammatory factors of the hippocampal tissues were examined by quantitative real‐time polymerase chain reaction (qRT‐PCR, *n* = 3). **(b)** The effects of Crocin on PI3K/AKT signaling and inflammation of hippocampal tissues in AD mice were examined by Western blot (*n* = 3). ^▲^ *p *< .05, ^▲▲^ *p *< .01 vs. sham group; ^★^ *p *< .05, ^★★^ *p *< .01 vs. Aβ_25‐35_ group.

The results of qRT‐PCR revealed that exposure to Aβ_25‐35_ caused obvious inflammation, as evidenced by the significant upregulation of IL‐1β, IL‐6, and TNF‐α mRNA expressions. However, Crocin treatment significantly reversed these effects (Figure 3a, *p *< .05). The results of Western blot analysis were consistent with those observed by qRT‐PCR (Figure 3b). In addition, Western blot analysis found that the phosphorylation of PI3K and Akt was decreased in the Aβ_25‐35_ group. Nevertheless, Crocin appeared to increase the phosphorylation of PI3K and Akt in AD mice (Figure 3b, *p *< .05).

## 4 DISCUSSION

Crocin is the primary constituent of *Crocus sativus* L. extract, which can be absorbed into the blood, and most of it will be converted into Crocetin for absorption, scholars have reported that the biological half‐life of Crocin is 2.67 h t1/2(α) and 81.53 h t1/2(β), indicating rapid distribution but relatively slow elimination (Ruiming, 1988; Wang et al., 2022). Mounting evidence has suggested that Crocin is effective in managing multiple diseases, including neurodegenerative diseases. For example, Wang et al. (2019) have suggested that Crocin can improve memory deficiency in mice with AD induced by d‑galactose and aluminum trichloride by using the MWM test. Another research has clarified that Crocin can ameliorate memory deficiency by inhibiting Aβ‐induced apoptosis and oxidative stress (Asadi et al., 2015). Furthermore, Crocin has been reported to enhance the memory abilities of aging Wistar rats by exerting its antioxidant activities (Heidari et al., 2017). Although few studies definitively state whether Crocin can cross the blood‐brain barrier, several studies have demonstrated that Crocetin can cross the blood‐brain barrier (Shahbaz et al., 2022). In addition, Zhang et al. (2017) have reported that Crocin may protect against cerebral ischemia in elderly patients by maintaining the integrity of the blood‐brain barrier. In this study, the classical methods of the OFT test, Y‐maze test, and MWM test were exploited to evaluate the behavior ability of AD model animals (Brandeis et al., 1989; Jiang et al., 2023). Consistent with previous reports, the present study also found that Crocin notably improved the cognitive deficits of Aβ_25‐35_‐induced AD mice.

Aβ is an inflammatory stimulator, and the accumulation of Aβ can activate glial cells, which further leads to the excretion of various inflammatory factors, thereby creating a proinflammatory environment that can compromise neuronal integrity (Hampel et al., 2021; Jun et al., 2023). According to reports, Aβ deposition and metabolic disorder are central links to the occurrence and development of AD, and abnormal deposition of Aβ can activate microglia and astrocytes and induce neuroinflammation, thus accelerating the pathogenesis of AD and exacerbating cognitive and memory disorders in patients (Guedes et al., 2018). Additionally, it has been reported that anti‐neuroinflammation can alleviate cognitive impairment and improve spatial learning and memory deficiency in AD animals (Cai et al., 2019; Sun et al., 2020). Crocin has been found to have anti‐neuroinflammatory effects in previous studies. For instance, one study has proposed that Crocin effectively impedes LPS‐mediated microglial activation and decreases the expression of proinflammatory mediators (IL‐1β and TNF‐α) (Lv et al., 2016). Furthermore, some inflammatory mediators, such as TNF‐α, IL‐1β, and IL‐6, play a significant role in the development of epilepsy, and Crocin presents an inhibitory effect on these inflammatory cytokines (Wang et al., 2017). Song et al. (2022) have reported that Crocin mitigates cognitive impairment related to atherosclerosis by impeding neuroinflammation. By conducting Western blot and qRT‐PCR analysis, we found that exposure to Aβ_25‐35_ resulted in an excessive inflammatory response, as evidenced by a significant increase of TNF‐α, IL‐6, and IL‐1β production. These findings suggested that Aβ_25‐35_ may contribute to the occurrence of neuroinflammation. However, Crocin suppressed the release of inflammatory factors in the hippocampus tissues of Aβ_25‐35_‐induced mice, concomitant with the downregulation of TNF‐α, IL‐6, and IL‐1β, proving that Crocin displayed an anti‐neuroinflammatory role in AD. Thus, we speculated that Crocin ameliorated spatial learning and memory deficiency in AD mice, possibly due to its anti‐neuroinflammatory effects.

The PI3K/AKT pathway displays a crucial role in the behavioral performance and learning and memory functions of rodents (Ali et al., 2018). This pathway is closely related to cellular survival, metabolism, and apoptosis, and dysregulation of this pathway will lead to various diseases, including dementia, tumors, and diabetes (Wang et al., 2010). Meanwhile, the upregulation of the PI3K/AKT pathway can modulate different downstream kinases, such as GSK‐3β or FOXO3a, to regulate the microglial phenotype and inhibit the activation of inflammatory signals in microglia, thereby improving neuroinflammation (Wang et al., 2020; Yang et al., 2020). In addition, the PI3K/AKT pathway can also modulate the synaptic plasticity of neurons, which means that cognitive ability can be improved by activating the PI3K/AKT pathway (Cui et al., 2017). Crocin has been found to suppress oxidative stress and neuroinflammation in microglial cells associated with diabetic retinopathy by activating the PI3K/AKT pathway (Yang et al., 2017). Crocin also has been found to alleviate depression resulting from chronic obstructive pulmonary disease by modulating PI3K/AKT‐mediated inflammatory pathways (Xie et al., 2019). This study clarified the anti‐neuroinflammatory and the amelioration of cognitive impairment of Crocin in Aβ_25‐35_‐induced mice, all of which were modulated via the activation of PI3K/AKT signaling. This study investigated the protective effects of intraperitoneal injection of Crocin on the learning and memory ability of AD mice. It is necessary to further analyze the effects of different doses and administration times on the cognitive and memory impairment of AD mice.

Together, our results indicated the neuroprotective effects and molecular mechanisms of Crocin in AD mice. Specifically, Crocin treatment suppressed inflammation by activating the PI3K/AKT signaling, which led to an improvement in the cognitive dysfunction of AD mice. This study laid a foundation for further exploration of the protective effects of Crocin on AD.

## AUTHOR CONTRIBUTIONS

**Wenwen Su**: Conceptualization; data curation; investigation; writing—original draft. **Yanbo Wang**: Investigation; methodology. **Sen Shao**: Methodology; software; data curation. **Xiaojun Ye**: Writing—review and editing; investigation; data curation.

## CONFLICT OF INTEREST STATEMENT

There are no competing interests between the authors.

### APPROVED THE VERSION TO BE PUBLISHED: ALL AUTHORS

Agreed to be accountable for all aspects of the work in ensuring that questions related to the accuracy or integrity of any part of the work are appropriately investigated and resolved: Wenwen Su, Yanbo Wang, Sen Shao, and Xiaojun Ye.

### PEER REVIEW

The peer review history for this article is available at https://publons.com/publon/10.1002/brb3.3503.

## Data Availability

The datasets generated during and/or analyzed during the current study are available from the corresponding author upon reasonable request
